# Aquaporins: The renal water channels

**DOI:** 10.4103/0971-4065.43687

**Published:** 2008-07

**Authors:** S. K. Agarwal, A. Gupta

**Affiliations:** Department of Nephrology, All India Institute of Medical Sciences, New Delhi - 110 029, India

**Keywords:** Aquaporins, diabetes insipidus, kidney, water channels

## Abstract

Water is the most abundant molecule in any cell. Specialized membrane channel, proteins called aquaporins, facilitate water transport across cell membranes. At least seven aquaporins (*AQP*): 1, 2, 3, 4, 6, 7, and 11 are expressed in the kidneys. Aquaporins play a role in both the short-term and long-term regulation of water balance as well as in the pathophysiology of water balance disorders. Aquaporin is composed of a single peptide chain consisting of approximately 270 amino acids. Inherited central and nephrogenic diabetes insipidus are primarily due to the decreased expression of *AQP2* while mutation in the *AQP2* molecule is responsible for inherited central diabetes insipidus. In acquired causes of nephrogenic diabetes insipidus, there is a downregulation of *AQP2* expression in the inner medulla of the kidney. Nephrotic syndrome is characterized by excessive sodium and water reabsorption, although in spite of this, patients do not develop hyponatremia. There is a marked downregulation of both *AQP2* and *AQP3* expression, which could be a physiologic response to extracellular water reabsorption in patients with nephrotic syndrome. There are some conditions in which aquaporin expression has been found to increase such as experimentally induced heart failure, cirrhosis, and pregnancy. Some drugs such as cisplatin and cyclosporine, also alter the expression of aquaporins. The three-pore model of peritoneal transport depicts the importance of aquaporins. Thus, the understanding of renal water channels has solved the mystery behind many water balance disorders. Further insights into the molecular structure and biology of aquaporins will help to lay a foundation for the development of future drugs.

Water is the most abundant molecule in a cell. Although the plasma membrane separates the interior of the cell from its extracellular environment, specialized membrane channels facilitate water transport across these biomembranes. Such water channels, which are in the form of proteins, are called aquaporins. In bacteria, plants, and animals, these channel proteins are very conserved, however, there are many human isoforms. At least seven aquaporins (*AQP*): 1, 2, 3, 4, 6, 7, and 11 are expressed in the kidneys [Table 1]. Aquaporins play a role in the short-term and long-term regulation of water balance and also in the pathophysiology of water balance disorders.

**Table 1:** Aquaporin distribution in the kidney

Aquaporin group	Localization in kidney
AQP 1	APM/BLM of proximal tubules and descending thin limbs
AQP 2	APM/VES of principal cells of collecting ducts
AQP 3	BLM of collecting ducts
AQP 4	BLM of medullary collecting ducts
AQP 6	Cortex, Medulla
AQP 11	Proximal tubule (Intracellular)

AQP: Aquaporin, APM: Apical membrane, BLM: Basolateral membrane, VES: Vesicles

## Discovery

Serendipity played a major role in the discovery of aquaporins. A 28 kDa polypeptide was noted during the biochemical purification of the 32 kDa core polypeptide of red cell Rh blood group antigen. The protein was found to be a tetramer and was functionally related to the major intrinsic protein of the lens.^1^ Abundance of the 28 kDa polypeptide in highly water permeable tissues, red cells, renal proximal tissues, and descending thin limbs led the late John C. Parker at the University of North Carolina at Chapel Hill to predict that it was probably a water channel. Peter Agre named the protein “aquaporin”.^2^

## Structure

**Fig. 1:**
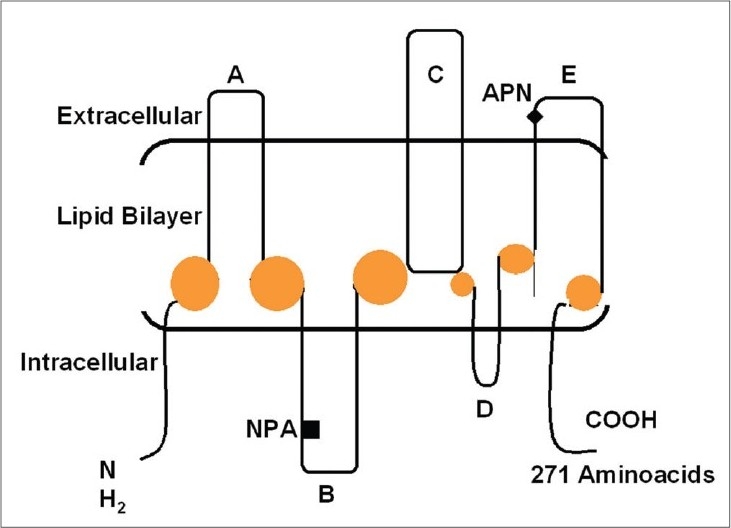
Structure of aquaporin 1

Aquaporin is composed of a single peptide chain consisting of approximately 270 amino acids. The deduced amino acid sequence of *AQP1* predicted six membrane-spanning domains with intracellular amino (*N*) and carboxy (*C*) termini. There are three extracellular loops (A, C, and E) and two intracellular loops (B and D). Highly conserved regions are present within loops B and E, each of which contains the consensus motif, asparagine-proline-alanine (NPA). When these two loops are folded into the lipid bilayer and surrounded by transmembrane domains, they may form a hydrophilic path for the water transfer through the lipid bilayer. This model is called the “hourglass model” [Fig. 1].^3^ Fourier transform infrared spectroscopy was used to further characterize the secondary structure of *AQP1* and the results revealed that six closely associated alpha helices span the lipid membrane.^4^ Moreover, the 3D structure of *AQP1* was determined at 6Å resolution by cryoelectron microscopy. Each *AQP1* monomer has six-tilted, bilayer-spanning alpha helices, which form a right-handed bundle surrounding a central density. These studies also confirmed the organization of a tetrameric complex in the membrane.^5^

## Aquaporin 1

The human aquaporin gene is located on chromosome 7p14. It has a strong homology with the major intrinsic protein of the bovine lens (MIP, *AQP0*) and was named initially as “channel-like integral protein of 28 kD”(CHIP-28) and later, Aquaporin 1 (*AQP1*). AQP1 is present in the apical and basolateral surfaces of the proximal convoluted tubules, the descending thin limbs of the loop of Henle, and in the nonfenestrated endothelium of the descending vasa recta.^6^ The localization of *AQP1* is in concordance with the known water permeability characteristics of the kidney, supporting the hypothesis that *AQP1* is mainly responsible for osmotic permeation in the regions of the kidney. It comprises 3.8% of the isolated proximal tubule brush border protein. As *AQP1* is absent in the collecting duct where water absorption is regulated by the antidiueretic hormone (ADH), *AQP1* must be responsible for constitutive water reabsorption.

In order to analyze the role of *AQP1* in water absorption for urine formation, *AQP1* null mice were generated by targeted gene disruption. The osmotic water permeability in the proximal tubule membrane vesicles is reduced by eight-fold compared to wild mice.^7^ This shows that *AQP1* is indispensable for efficient urine concentration, which is further confirmed by the partial correction of the urine-concentrating defect in *AQP1* null mice by introducing the *AQP1* via adenovirus-mediated gene delivery. These results also establish the cellular mechanism of water reabsorption: water passes through the epithelial layer not paracellularly, but transcellularly via the aquaporins. The Colton blood group antigen was identified to be an *AQP1* in human RBCs. Individuals lacking the Colton antigen showed *AQP1* gene nonsense / missense mutations, although they did not suffer from any clinical abnormalities.^8^ Recently, defective urinary-concentrating ability was shown in an individual completely lacking *AQP1*.^9^

## Aquaporin 2

**Fig. 2:**
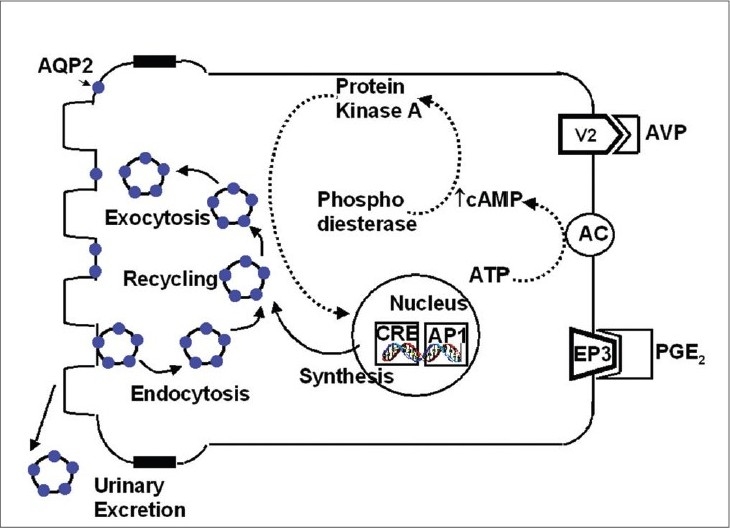
Regulation of aquaporin 2 in principal cell

*Aquaporin 2 (AQP2)* was cloned as an ADH-regulated water channel of kidney collecting ducts (CD) in rats and humans; it is present in the principal cells of the CD. In the basal state, *AQP2* is stored in intracellular vesicular compartment but upon ADH stimulation, it rapidly moves to the apical membrane where it acts as a water channel for the concentration of urine. Regulation of *AQP2* trafficking is shown in Fig. 2. Vasopressin acts at V2 receptors in the basolateral plasma membrane (BLM) of CD principal cells. Activation of adenyl cyclase accelerates the production of cyclic AMP from ATP; the cyclic AMP then binds to the regulatory subunit of protein kinase A (PKA) which activates the catalytic subunit of PKA. PKA then phosphorylates *AQP2* in the intracellular vesicles and possibly other cytosolic or membrane proteins. Microtubular motor proteins and vesicle targeting receptors (VAMP-2, Syntaxin-4, and NSF) may participate in the specificity of *AQP2* targeting to the apical membrane to increase water permeability. Cyclic AMP also participates in the long-term regulation of *AQP2* by increasing the levels of the catalytic subunit of PKA in the nuclei, which phosphorylates transcription factors such as cAMP responsive element-binding protein (CREB-P) and c-jun / c-fos. Binding of these proteins is thought to increase the gene transcription of *AQP2* resulting in synthesis of *AQP2* protein, which in turn, enters the regulated trafficking system.^10^

## Aquaporin 3

Aquaporin 3 (*AQP3*) is localized along the BLM of the principal cells of the collecting ducts. Along with *AQP2*, it aids in the exit of water from the cell into the interstitium for transepithelial transfer of water across CD cells. It permits transport of both water and glycerol and there is a possibility of separate water- and glycerol-transporting domains. Due to its ability to mediate glycerol transport, AQP3 was also called the glycerol intrinsic protein [GLIP]. Although the role of *AQP3* in water transport is not fully understood, an *AQP3* knockout experiment provided a clue. The growth and phenotype of *AQP3* null mice were grossly normal except for polyuria. These animals consumed ten times more fluid and excreted urine of low osmolality.^11^ No humans have yet been identified with *AQP3* deficiency.

## Aquaporin 4

Aquaporin 4 (*AQP4*) together with *AQP3* is localized at the BLM of the principal cells of CDs. It may serve as a water channel for the exit of water from the BLM for the concentration of urine. *AQP3/AQP4* double knockout mice show greater impairment of urine-concentrating ability than *AQP3* single knockout mice.^11^

## Aquaporin 6

Aquaporin 6 (*AQP6*) is expressed in the acid-secreting intercalated cells of the collecting ducts in the kidney. It co-localizes with H^+^ ATPase, suggesting that low pH could activate the protein. Intracellular vesicles in the α-intercalated cells are thought to permit renal acid secretion by generating hydrochloric acid. To maintain electroneutrality, a pathway for anion secretion must exist and ClC-5 is located at this site. However, this protein is inactivated at low pH. Thus, the role of *AQP6* in the α-intercalated cells may be to function as a chloride channel during the later stages of acid secretion.^12^

## Aquaporin 11

Although aquaporin 11 (*AQP11*) can be identified on the basis of the unusual pore forming asparagine-proline-alanine (NPA) consensus motif, its function is unknown. It is localized intracellularly in the proximal tubule. *AQP11* null mice exhibited vacuolization and cyst formation in the proximal tubule. *AQP11* null mice were born normally but died before weaning due to advanced renal failure with polycystic kidneys. Further culture revealed an endosomal acidification defect in *AQP11* null mice. This illustrates that *AQP11* is essential for proximal tubular function.^13^

## Pathophysiologic Roles of Aquaporins in Renal Disorders [Table 2]

**Table 2:** Water balance disorders

Conditions with reduced *AQP2* expression and polyuria
Central diabetes insipidus
Aging
Lithium
Low-protein diet
Hypokalemia
Hypercalcemia
Postobstructive nephrogenic diabetes insipidus
Ischemia-induced acute kidney injury
Experimental chronic renal failure
Conditions with increased AQP2 expression and water retention
Cardiac failure
Pregnancy
Cirrhosis

*AQP2:* Aquaporin 2

### Inherited central and nephrogenic diabetes insipidus

Central Diabetes insipidus (CDI) is characterized by very low or undetectable levels of vasopressin. Using vasopressin-deficient Brattleboro rats as a model, it was demonstrated that *AQP2* expression levels were markedly lower than those in the parent strains of Long Evans rats.^14^ Furthermore, prolonged treatment with either vasopressin or dDAVP results in marked increases in *AQP2* expression and in apical plasma labeling in both the inner medulla and the cortical collecting ducts. Thus, dysregulation of *AQP2* due to the absence of vasopressin plays a major role in the development of polyuria. In nephrogenic Diabetes insipidus (NDI), there is insensitivity to vasopressin in renal tubular cells.

90% of the inherited types are X-linked recessive due to mutations in the *V2 receptor* genes. The rest are either autosomal dominant or autosomal recessive mutations in the *AQP2* gene located on chromosome 12q13.^15^

In the autosomal recessive type, mutations are mostly between the 5^th^ and 6^th^ transmembrane domains of the molecule. Two types of mutations have been identified: T125M or G175R with normal trafficking to plasma membrane but leading to the loss of water channel function, and another one that causes misrouting of the mutated *AQP2* molecule to the endoplasmic reticulum leading to a failure to reach the plasma membrane. This misfolding can be corrected by chemical chaperones. In the autosomal dominant form, mutations are found in the C-terminus of *AQP2*. This mutated *AQP2* is localized in aberrant intracellular compartments such as the Golgi apparatus, lysosomes, or BLM. Here, trafficking of *AQP2* to the plasma membrane is impaired, but its fundamental 3-D structure is not affected by these mutations.^16^,^17^

### Acquired causes of nephrogenic diabetes insipidus

Hypokalemia and hypercalcemia may be associated with polyuria as a result of a defect in the renal response to ADH to maximally concentrate urine. A potassium-deficient diet fed to rats for 11 days caused hypokalemia-related nephrogenic diabetes insipidus (NDI) and seven days of a vitamin D-rich diet caused hypercalcemia-related NDI. There was downregulation of *AQP2* expression in the inner medulla in both these studies.^18^,^19^ Chronic lithium treatment resulted in a severe concentration defect and in the rat model, lithium caused a marked reduction in *AQP2* levels in parallel with the development of severe polyuria. Urine volume increased three-fold after one week of lithium treatment and > six-fold after two weeks while *AQP2* expression decreased to 58% of that in control rats at one week and to 33% at two weeks of lithium treatment. With the aid of proteomics, 77 different proteins were identified within the inner medullary collecting duct that were affected directly or indirectly by lithium treatment.^20^ Another cause of acquired NDI is postobstructive diuresis. Bilateral ureteral obstruction in a rat model showed that *AQP2* levels were reduced to ¼^th^ of the control levels. Even one week after the urine output had become normal, animals showed only ½ of their normal *AQP2* levels. Secondly, they could not concentrate urine in response to dehydration as effectively as sham-operated rats.^21^

A low-protein di *et al*so causes a urine concentration defect. In rats fed on a low-protein (8%) diet for two weeks, *AQP2* expression decreased significantly in the inner medulla with a subsequent decrease in ADH-stimulated osmotic water reabsorption.^22^ Aging decreases the ability to elevate ADH levels in response to dehydration. A study revealed that 72 hours of water deprivation in four month- and 30 month-old rats resulted in increased *AQP2* levels in only the younger rats.^23^

### Aquaporin dysregulation in renal diseases

Nephrotic syndrome is characterized by excessive sodium and water reabsorption, but in spite of this, patients do not develop hyponatremia. There is marked downregulation of *AQP2* and *AQP3* expression, which could be a physiological response to extracellular water reabsorption.^24^ In ischemic ARF, structural changes are seen in the S3 segment of the proximal tubule and the thick ascending limb. Recently, Fernandez-Llama showed that CD water channels, *AQP2, 3, 4* as well as proximal tubule AQP1 expression levels were significantly decreased in association with polyuria in response to ischemia. This suggests that CD is also affected by ischemia and that the polyuria seen in ARF may be partially due to reduced CD aquaporin levels.^25^ Similarly, experimental CRF induced by 5/6th nephrectomy is characterized by polyuria and ADH-resistant downregulation of *AQP2* and *3* levels.^26^

Normally aquaporin is present in minimal amounts in the glomerulus. Immunohistochemistry of biopsy samples showed marked upregulation of *AQP1* in the glomeruli in various renal disorders. Bedford *et al.* postulated that renal injury produced increased stress on cell integrity and increased aquaporin expression is an adaptive response to this stress.^27^ In contrast, they found a reduction in the expression of *AQP2* and *3* in lesions with substantial interstitial fibrosis and reduced nephron numbers.

### Conditions with increased aquaporin expression

Heart failure experimentally induced by left coronary artery ligation in rats showed that there was marked increase in *AQP2* mRNA expression and a redistribution of *AQP2* in the collecting ducts with increased targeting to the apical membrane. This regulation was seen only in rats with severe CHF (increased LVEDP and decreased serum sodium) but not in rats with compensated failure (increased LVEDP and normal serum sodium). Cirrhosis is another condition with variable *AQP2* expression. Chronic intraperitoneal administration of carbon tetrachloride results in increased *AQP2* mRNA and protein in rats. The V2 receptor antagonist, OPC 31260 reversed this increase. However, compensated cirrhosis induced by ligation of the common bile duct resulted in decreased *AQP2* expression and consequently, V2 receptor antagonists were not effective. Another condition is pregnancy where rats had increased *AQP2* levels in the first trimester that persisted throughout pregnancy; increased *AQP2* trafficking was also seen. V2 receptor antagonists were effective in reducing *AQP2* levels to those seen in the nonpregnant state.^28^

### Aquaporins and drugs

In a controlled study, cisplatin (8 mg/kg) was injected intraperitoneally into male Sprague-Dawley rats. Four days later, the expression of *AQP1, AQP2*, and *AQP3* proteins was determined in the kidney. Cisplatin treatment caused a polyuric renal failure in association with decreased free water reabsorption. Expression of *AQP1* and *AQP2* was decreased in the cortex and the outer and inner medulla, whereas that of *AQP3* was decreased in the outer and inner medulla.^29^ In another study, rats were treated daily for four weeks with vehicle (VH; olive oil, 1 mL/kg *sc*) or cyclosporine, CsA (15 mg/kg *sc*). Increased urine volume, fractional excretion of sodium, decreased urine osmolality, and free-water reabsorption was seen in the CsA group as compared to the VH-treated rats. There was a decrease in the expression of *AQP* (1–4) and the urea transporter, UT (UT-A2, -A3, and UT-B). This shows that calcineurin is required for normal intracellular trafficking of *AQP2*; loss of calcineurin protein or activity disrupts *AQP2* function.^30^

### Aquaporins and peritoneal dialysis

**Fig. 3:**
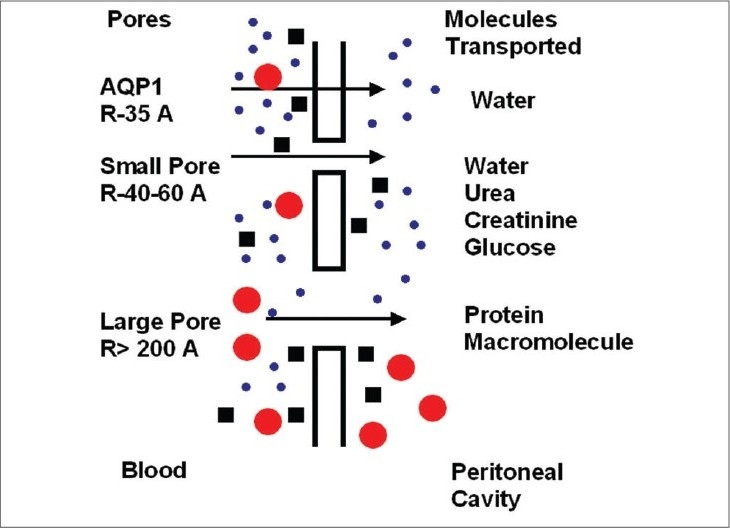
Three-pore model of peritoneal transport

The three-pore model of peritoneal transport depicts the importance of aquaporins. It assumes the capillary endothelium to be the major barrier to solute and water transport occurring through a system of pores that are classified into three broad categories: ultrasmall, small, and large pores. The abundant small pores (40 to 60 Å radii) are the tortuous intercellular clefts between the endothelial cells; they are responsible for small solute transport. The ultrasmall pores (radius 3 to 5 Å), also present in large numbers, are probably the transendothelial aquaporin-1. Solute free-water transport occurs across them by way of crystalloid osmosis. In addition, a few large pores (200 to 300 Å radii) are present, the nature of which is not well known; macromolecules such as albumin are transported across them [Fig. 3].

It is assumed that 40–50% of osmotic-induced ultrafiltration is mediated by aquaporins. As free water is transported through these pores, a drop in dialysate sodium concentration is expected to occur during the initial 60 to 90 minutes of the dwell. This phenomenon is known as sodium sieving. A dialysate/plasma sodium ratio of >0.88 correlates with an aquaporin deficiency at 60 minutes during the modified peritoneal equilibrium test.

## Conclusion

The recognition and understanding of renal water channels has solved the mystery of many water balance disorders. Further insights into the molecular structure and biology of aquaporins will lay a foundation for the development of future drugs.
